# N-terminal pro-brain natriuretic peptide levels during the acute phase of sepsis may be a useful indicator of higher risk of long-term impairments: some confounders to consider

**DOI:** 10.1186/s13054-020-2820-z

**Published:** 2020-03-17

**Authors:** Patrick M. Honore, Cristina David, Aude Mugisha, Rachid Attou, Sebastien Redant, Andrea Gallerani, David De Bels

**Affiliations:** ICU Department, Centre Hospitalier Universitaire Brugmann-Brugmann University Hospital, Place Van Gehuchtenplein, 4, 1020 Brussels, Belgium

Custodero et al. conclude that N-terminal pro-brain natriuretic peptide (NT-proBNP) levels during the acute phase of sepsis may be a useful indicator of higher risk of long-term impairments in physical function and muscle strength in sepsis survivors [1]. A letter from Jiarong et al. has challenged this assertion, pointing to the exponential increase in the plasma level of NT-proBNP with a declining glomerular filtration rate. As they have noted, it does not seem persuasive that NT-proBNP could completely predict outcomes without adjusting for the covariate of renal function. They suggest that the relationship of NT-proBNP levels during the acute phase of sepsis and physical function and muscle strength outcomes in sepsis survivors be stratified based on the renal function [2]. In keeping with this, we would like to comment further. Nearly half of critically ill patients, especially with septic shock, have or develop acute kidney injury (AKI) and 20–25% need renal replacement therapy (RRT) within the first week of their stay [3]. In the Custodero study, the two cohorts (chronic critical illness [CCI] and rapid recovery [RAP]) had a considerable difference in the incidence of septic shock (36.5% vs 16.4%), so it would stand to reason that the rate of AKI and continuous renal replacement therapy (CRRT) was much lower in the RAP cohort when compared to the CCI cohort [1]. CRRT is performed using membranes that have a cut-off value of 35–40 kDa and therefore some quantity of NT-proBNP will be eliminated [4]. Because of its low molecular weight (8.5 kDa), NT-proBNP is likely to be effectively cleared by both high- and low-flux membranes [4]. New highly adsorptive membranes (HAM) can adsorb many molecules with a molecular weight above 35 kDa and will increase this removal even further [4]. This could mislead patient prognostication by artificially decreasing NT-proBNP, but no studies have challenged this issue. Such studies should be done as there is already a long list of biomarkers in sepsis that are lacking reliability during CRRT [5]. As a consequence of the different rates of CRRT between the two cohorts, the reliability of NT-proBNP to be a useful indicator of long-term impairments in physical function and muscle strength in sepsis survivors might be questioned.

## Authors’ response

Carlo Custodero; Quran Wu; Gabriela L. Ghita; Stephen D. Anton; Scott C. Brakenridge; Babette Brumback; Philip Efron; Anna K. Gardner; Christiaan Leeuwenburgh; Lyle L. Moldawer; John W. Petersen; Frederick A. Moore; Robert T. Mankowski

We highly appreciate the feedback from Honore et al. on our publication entitled **“**Prognostic value of NT-proBNP levels in the acute phase of sepsis on lower long-term physical function and muscle strength in sepsis survivors.”

We agree that impaired renal function is one of the factors contributing to high NT-proBNP levels in the acute phase of sepsis [6]; however, we addressed this comment in our previous response by showing that adding the estimated glomerular filtration rate (eGFR) as a covariate to our model did not change our findings. We agree that the chronic critical illness (CCI) patients had higher rate of acute kidney injury (AKI) and that the levels of NT-proBNP can be affected by therapies such as the continuous renal replacement therapy (CRRT) [4]. However, in our cohort, only 12% of CCI and 0% of RAP patients had CRRT within 48 h from sepsis onset.

Our primary purpose of showing this predictive association was to continue this line of research to further understand the biology of high NT-proBNP levels in acute phase of sepsis and as a novel approach to potentially identify sepsis patients at high risk for functional decline. Therefore, we agree with the authors that other factors such as CRRT in artificially decreasing the NT-proBNP levels should be taken into consideration in our future investigations to further explore the biological factors that may underlie high NT-proBNP levels in the acute sepsis patient population.

## Data Availability

Not applicable.

## Abbreviations

NT-proBNP: N-terminal pro-brain natriuretic peptide
CCI: Chronic critical illness
RAP: Rapid recovery
ICU: Intensive care unit
AKI: Acute kidney injury
RRT: Renal replacement therapy
CRRT: Continuous renal replacement therapy
HAM: Highly adsorptive membranes
